# Bibliography

**Published:** 1905-08

**Authors:** 


### Bibliography.

Notes on Dental Porcelain. A Practical Treatise especially devoted to the Interests of the Beginner. By V. Walter Gilbert, D.D.S. Over one hundred practical illustrations. The S. S. White Dental Manufacturing Company, Philadelphia; Claudius Ash & Sons, Limited, London, 1905.

History constantly repeats itself, but assumes new forms at every repetition. This is being demonstrated in dentistry at almost every recurring decade. This fact was impressed upon the writer upon taking up Dr. Gilbert's book for review, and his mind reverted to a period when the knowledge of dental porcelain was an absolute necessity for any one attempting the practice of dentistry. Then came a long interregnum in which the knowledge of dental porcelain was confined to the manufacturers of porcelain teeth, and now the eternal cycle of thought has brought the student of dentistry, as well as practitioners, face to face with the fact that he must learn, willingly or unwillingly, the manipulation of porcelain bodies as his fathers did before him.

It is, therefore, very appropriate that a book should have been written by one combining a scientific and practical training in one of the largest dental schools of the country with a very exact knowledge of porcelain through his intimate connection with one of the largest, if not the largest, manufactures of dental porcelain of the world. All the experience accumulated in this house since the days of Stockton have been at the author's command, and that he has made good use of this knowledge is apparent throughout the book.

There has been so much written, of a seemingly partisan character, by those engaged in the modern use of porcelain in practice, that it is not surprising that the novice is in a state of embarrassment as he hears and reads of "high fusing" and "low fusing," until he questions the value of either and naturally fears to attempt this material in any form, or, as the author states it, "The opinion of the high-fusing enthusiast is accepted only to be discarded upon reading or hearing the opinion of the low-fusing enthusiast, and *vice versa.*"

The author, very properly, begins his first chapter on the consideration of "Porcelain: Its Place in Dentistry." Its place is so important that, while dentistry existed prior to the advent of porcelain in tooth forms, it was not dentistry as understood to-day, and the author very forcibly places this before his readers, wherein he imagines the condition if "All the artificial teeth, porcelain bodies, gum enamels, etc., were destroyed and no longer obtainable." Where, indeed, would dentistry be were such to happen? Prosthetic dentistry is dependent upon porcelain. Operative dentistry is becoming more and more subject to its artistic influence, and the world at large is dependent upon it for mastication of food, health, and even length of life.

There is so much to learn regarding porcelains, that it has become a serious question whether the schools will not be forced to go back to the work of the Fathers and teach the entire process. The author expresses the importance of this knowledge when he writes: "Do not make an initial mistake by supposing that any one grade of porcelain may be regarded as a 'cure all' and that it possesses qualities which will meet all dental requirements. . . . A porcelain compound which has been especially prepared to meet the requirements of a full artificial denture will not be entirely satisfactory for the production of a small inlay. A porcelain compound especially prepared to meet the requirements of a small inlay will not be entirely suitable for the production of a full artificial denture." These are wise words, and should be carefully considered by those who rush from an inlay to the making of a crown, or crowns, entirely of porcelain.

The confusion of terms, "High Fusing" and "Low Fusing," which really mean nothing, but which the novice in porcelain work regards as defining a definite quality, places the operator in an embarrassing position. The author attempts to remedy this by defining the meaning which should be placed on these terms. He says, "In order to make the term a little more comprehensive to the student, the generally accepted definition of 'High Fusing' will be modified to read as follows: A porcelain which vitrifies or takes the glaze at a temperature somewhere between the melting point of pure gold and the glazing point of an American-made porcelain tooth;" and "Low Fusing" is applied to "compounds which vitrify or take the glaze at a temperature below the melting point of pure gold."

The author's chapter on "Laboratory experiments" is an exceedingly interesting one, but aside from this it is of very decided practical value. In order to test the "differences in fusing point," ten of the best known products were taken and subjected, in the form of cones of porcelain, to a heat sufficient to glaze. The result demonstrated the effect of heat upon the different bodies, melting absolutely the low-fusing, and proportionately so with the others, eventually leaving one cone to stand up against all the others of the high-fusing series. These experiments are not only novel in character, but demonstrate at a glance the futility of calling all the compounds by one name,—"High Fusing."

Space will not permit following this valuable book through all the chapters. These are written in a captivating style, that holds the reader to the end, at least such was the experience of the reviewer. The author has the faculty of making each operation clear, and the beginner who proposes to enter upon the preparation of porcelain in any of its dental forms will do well to procure this book, and in so doing will be able to work his way through a maze of many difficulties.

In order that our readers may have a general idea of the scope of this book of one hundred and twenty-six pages, the titles of chapters are appended: "Porcelain: Its Place in Dentistry. Dental Porcelains. Laboratory Experiments. Practical Application of Block Bodies. Porcelain Crowns. Porcelain Fillings. Shading Porcelain. Mineral Stains. Furnaces. Fusing Porcelains."

It is a gratification to find one of the younger men connected with dentistry ready to place his acquired knowledge in the hands of those struggling amidst many difficulties in the acquirement of a difficult art, but while it has been written with these principally in view, it will be found a very suggestive production for those older practitioners who may feel themselves well grounded in the work.

Messrs. Lea Brothers & Co. announce a new edition of "Gray's Anatomy," to be published about midsummer, and embodying nearly two years of labor on the part of the editor, J. Chalmers Da Costa, M.D., of Philadelphia, and a corps of special assistants.

Commensurately with the importance of the largest-selling medical work ever published, this new edition will present a revision so thorough and searching that the entire book has been reset in new type. In addition to the changes necessary to bring it abreast of the most modern knowledge of its subject, several important alterations have been made with the view of adapting it still more closely to present-day teaching methods, and, in fact, to anticipate the trend of anatomical work and study.

The illustrations have come in for their full share of the general revision, so that at this writing more than four hundred new and elaborate engravings in black and colors have been prepared. "Gray" has always been noted for its richness of illustration, but the new edition far exceeds anything that has hitherto been attempted.

A Manual of Mechanical Dentistry and Metallurgy. By Geo. W. Warren, A.M., D.D.S., Professor of Clinical Dentistry, Pennsylvania College of Dental Surgery; author of "A Compend of Dental Pathology and Dental Medicine," etc. Second edition, revised, with seventy-nine illustrations. P. Blakiston's Son & Co., Philadelphia.

This work of two hundred and fifty-seven pages has been greatly improved since the first edition was reviewed in this journal.

All manuals must be in a large degree unsatisfactory to the one fond of detail, and it may be doubted whether they are of much real benefit to the student. Their tendency, as a rule, seems to be to cultivate superficial knowledge at the expense of thoroughness. This the author has tried to avoid, and has measurably succeeded for the purpose intended,—that of giving a "conservative guide for the student in his study of the subjects considered." The author says, "It is presented as a convenient every-day working book," and in this sense it can be recommended as a work of reference in the laboratory.

Henry's Appointment-Register-Day-Book for Dentists. By Robert S. Henry, D.D.S., Chattanooga, Tenn.

This appointment-book is one of the most satisfactory of its kind. It covers every day from January to December, and with the proper date given on each page. Upon each page at its head is a diagram of the teeth in each jaw, anterior and posterior, with abbreviations to designate the various operations, and in addition Dr. and Cr. columns. The facility with which a record can be made in it of examinations constitutes its greatest recommendation, but it seems, in other respects, to cover all the requirements of an engagement-book for the daily work of the office.

